# Effect of Perchloryl Fluoride Additions on the Flame Speed of Methane

**DOI:** 10.6028/jres.065A.055

**Published:** 1961-12-01

**Authors:** Carl Halpern

## Abstract

The effects of addition of small quantities of perchloryl fluoride and of oxygen on the flame speed of methane-air mixtures have been determined and are compared with each other and with the effect of moderately preheating the burning mixture. Only small additions of perchloryl fluoride, up to 3 percent by volume, could be used because of the corrosiveness of its combustion products. Perchloryl fluoride is more effective than oxygen in increasing the flame speed of methane but less effective, in the amounts added, than preheating the burning mixture to 330°F. Maximum flame speeds for given experimental conditions were found to vary linearly with increasing additions of either perchloryl fluoride or oxygen.

## 1. Introduction

The determination of the effect on flame speed of small additions of perchloryl fluoride was carried on concurrently with another task in which gross effects in the combustion of hydrocarbons with perchloryl fluoride addition in a large duct were measured. Results of experiments in the duct reflected the combined influences of high temperature, variation of pressures, turbulence, and fuel-oxidant mixture ratio. Since closer control of conditions is possible in a small burner, the effects of the parameters considered on the flame speed are more clearly identified, and thus the information obtained is more basic. Methane was chosen as the fuel because we had had much experience in the determination of flame speeds of methane-air mixtures. At the conclusion of the experiments with perchloryl fluoride, a series of experiments was run with similar quantities of oxygen added to methane-air mixtures to compare the effects of oxygen and perchloryl fluoride.

Perchloryl fluoride (ClO_3_F) is a relatively new compound, discovered in 1952 [1].^1^ It is stated [2] to be an inert gas at room temperature, stable and noncorrosive when dry; its freezing point is − 146 °C and its boiling point is −46.8 °C. In the presence of water, it is very corrosive to most metals. Its heat of formation is −5.12 kcal/mole [3]; thus it can be expected, under the proper conditions, to be very active chemically, with high heats of reaction.

Lodwig and Margrave [4], in a study of perchloryl fluoride flames, have reported a few determinations of the flame speeds of mixtures of 65–75 percent methane and 35–25 percent perchloryl fluoride, by volume. Their values for flame speeds of these mixtures range from about 185 to 195 cm/sec (centimeters per second) and average about 190 cm/sec (6.2 fps) (feet per second). However, their method for determining flame speeds, which is based on the measurement of the area of the visible inner cone of a Bunsen flame, is not now considered to yield reliable values. The preferred method is considered to be that based on the measurement of the schlieren cone [5, 6].

## 2. Apparatus and Procedure

A description of the apparatus and the method used to measure flame speeds has been presented earlier [7]. Briefly, this apparatus comprises drying and metering systems for both air and fuel, a conditioning chamber for the mixture of fuel and oxidant, and a nozzle, the exit of which is the burner port. Means are provided to control the temperature of the combustible mixture issuing from the nozzle.

For the present experiments it was decided to prepare mixtures of air and the added oxidant (perchloryl fluoride or oxygen) of the desired strength and to meter this mixture, rather than to set up a third metering system. Some error in the measurement of the flow of these mixtures was inevitable, since the calibration of the sharp-edged orifice used to meter the gas depended on the density of the gas. However, in view of the small quantities of additive used (5 percent by volume maximum for perchloryl fluoride and 6 percent for oxygen), it was felt that the error so introduced would be tolerable. The mixtures of air and added oxidant were prepared in a 120 gal (16.05 cu ft) galvanized steel tank. The tank was evacuated to a pressure of several microns, and the oxidant was admitted from its container; pressure of the oxidant was measured by a mercury manometer, read to 0.01 in. Air from a compressor was dried by passing first through a column of activated alumina and then through a cold trap immersed in a slush of dry ice in a mixture of equal parts by weight of carbon tetrachloride and chloroform. It was then slowly admitted to the tank. Water content was thus kept to 0.03 percent by volume. The final pressure in the mixing tank was generally about 150 psig and was read on a calibrated Bourdon gage to 0.1 lb.

Because the products of combustion of perchloryl fluoride include hydrogen chloride and hydrogen fluoride, the exhaust gas was drawn by a large capacity vacuum pump from the enclosure surrounding the burner nozzle, and then through a chamber packed with copper turnings before being exhausted into the air.

Mixtures of 0.5, 1, 2, 3, 4, and 5 percent by volume of perchloryl fluoride in air were prepared. For each mixture, attempts were made to determine the variation of flame speed with mixture ratio, by weight, of methane to air plus added perchloryl fluoride, with the velocity of the gas at the port of the nozzle held constant. The mixture ratio ranged from 0.054 to 0.072 and the velocity of the gas mixture at the port of the nozzle ranged from 4 to 7 fps.

Burning mixtures prepared with methane and air containing 1 and 2 percent, respectively, by volume of perchloryl fluoride yielded stable flames over the entire range of mixture ratios and gas velocities used.

Table 1 lists the conditions at which blowoff and flashback were encountered with the other mixtures. Blowoff represents the lean limit of operation of the burner, at which flames cannot be maintained; flashback represents the rich limit of operation.

Formation of a white deposit on the lip of the port of the nozzle during combustion of methane with air-perchloryl fluoride mixtures interfered with the gas flow and resulted in unstable, misshapen flames. Hydrogen fluoride and hydrogen chloride in the mantle of hot gas surrounding the inner cone of the flame undoubtedly had reacted with the metal of the nozzle to form a mixture of chlorides and fluorides. This deposit of salts was soft and easily removed. Rate of deposition was slow with weak mixtures of perchloryl fluoride and air; with proper care, stable conical flames were maintained. Rate of deposition with 4 and 5 percent mixtures, however, was so fast that undistorted flames could not be maintained. Hence, flame speeds could not be determined.

In a zone beginning about 0.03 in from the lip of the port of the nozzle and extending for about 0.2 in, the metal was eaten away to a depth of about 0.02 in, leaving a thin rim of untouched metal surrounding the port of the nozzle. During the first experiments with the 3 percent mixture of perchloryl fluoride, this rim burned through at one spot, thus creating permanently distorted flames. Experimentation with perchloryl fluoride was discontinued and the nozzle was removed from the burner system for repairs. Of the six bronze screens used to smooth the gas flow in the conditioning chamber of the burner assembly, the two top ones were heavily corroded and had to be replaced. Since flashback of perchloryl fluoride flames had taken place several times, it was felt that the corrosion of the screens was a result of these occurrences. The other four screens were in good condition. The reflecting surface of the spherical mirror used to focus light on the schlieren stop was eaten away in spots; this mirror was also replaced. The sharp-edged orifice used to meter the mixtures of air and perchloryl fluoride was in good condition.

Mixtures of air and 0.5, 1, 2, 3, 4, 5, and 6 percent, by volume, of added oxygen were prepared. Variation of flame speed with mixture ratio, by weight, of methane to air plus added oxygen was determined for each mixture. Velocity of the effluent gas at the nozzle port was held constant. As in the case of perchloryl fluoride, mixture ratio varied from 0.054 to 0.072, and gas velocity ranged from 4 to 7 fps.

Burning mixtures made from air containing 3, 4, and 5 percent by volume of added oxygen gave stable flames over the entire range of mixture ratios and gas velocities used. Table 1 lists the conditions at which blowoff and flashback occurred with mixtures of other strengths. As mentioned above, these represent the lean and rich limits of operation for the burner.

The temperature controlled nozzle was used throughout these experiments, but the control of temperature was not as good as had been in previous experiments. Because of the limited supply of oxidant mixture, the nozzle could not be brought to thermal equilibrium, so there was a change in temperature during an individual run. The largest temperature change during a run of about three hours’ duration was a rise of nine degrees Fahrenheit; generally the temperature change did not exceed five degrees. Since the fluid circulating in the nozzle was cooled by tap water, temperature of the nozzle and hence of the incoming combustible mixture depended on the temperature of the tap water; as the experiments reported here lasted over a year, there was considerable seasonal variation in the nozzle temperature. The winter temperature was about 80–85 °F, while the summer temperature was about 95–100 °F. The variation of flame speed with mixture temperature for methane-air flames had been found to be 0.0048 ft/°F [7]. The effect of temperature on methane-air-perchloryl fluoride and methane-air-oxygen flames, especially at low concentration of additives, should be of similar magnitude. Over a temperature range of 15 °F, this would amount to about 0.07 fps. For a flame speed of about 1.5 fps, poor temperature control could introduce a variation of about 7 percent between summer and winter, and about 2 percent in an individual determination when the change of the temperature of the nozzle and of the incoming combustible mixture is 5 °F.

## 3. Results

**Figure 1:**
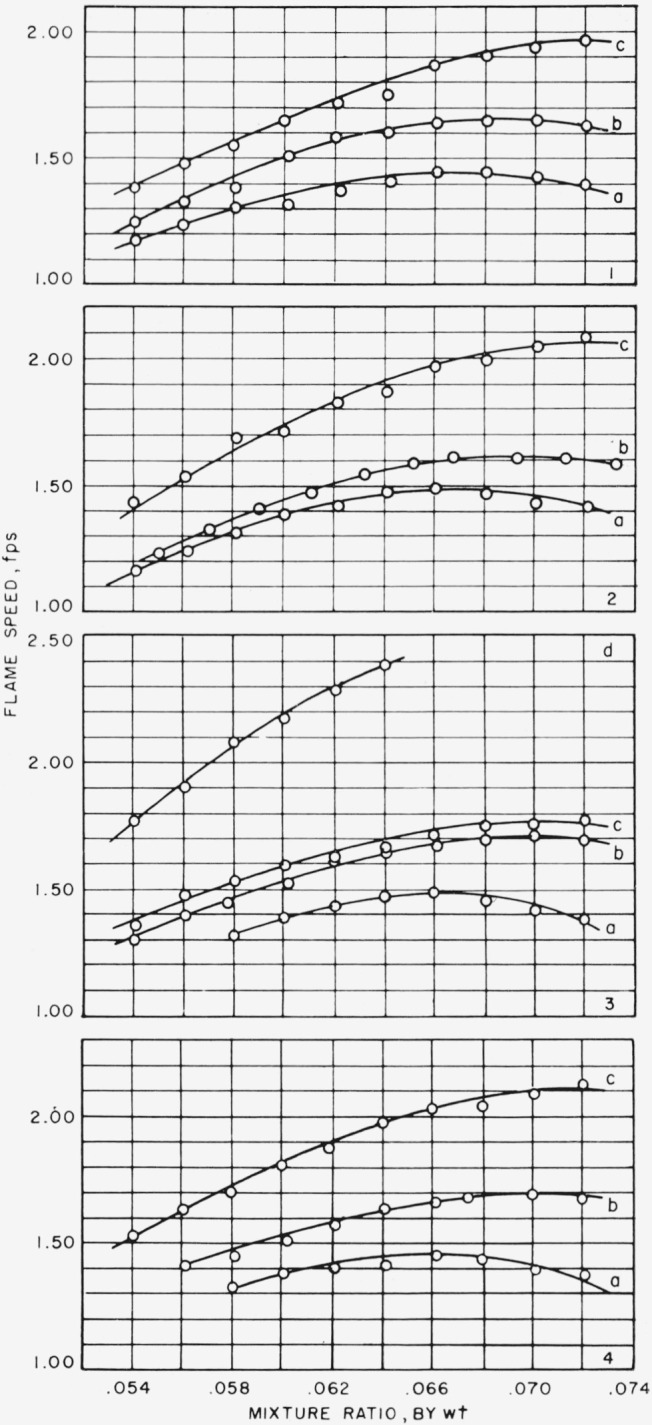
Effect of mixture ratio, by weight, of methane to air plus perchloryl fluoride on flame speed. Percent, by volume, of added perchloryl fluoride=a, 0.5; b, 1.0; c, 2.0; and d, 3.0. Gas velocity = 1, 4 fps; 2, 5 fps; 3, 6 fps; and 4, 7 fps.

**Figure 2:**
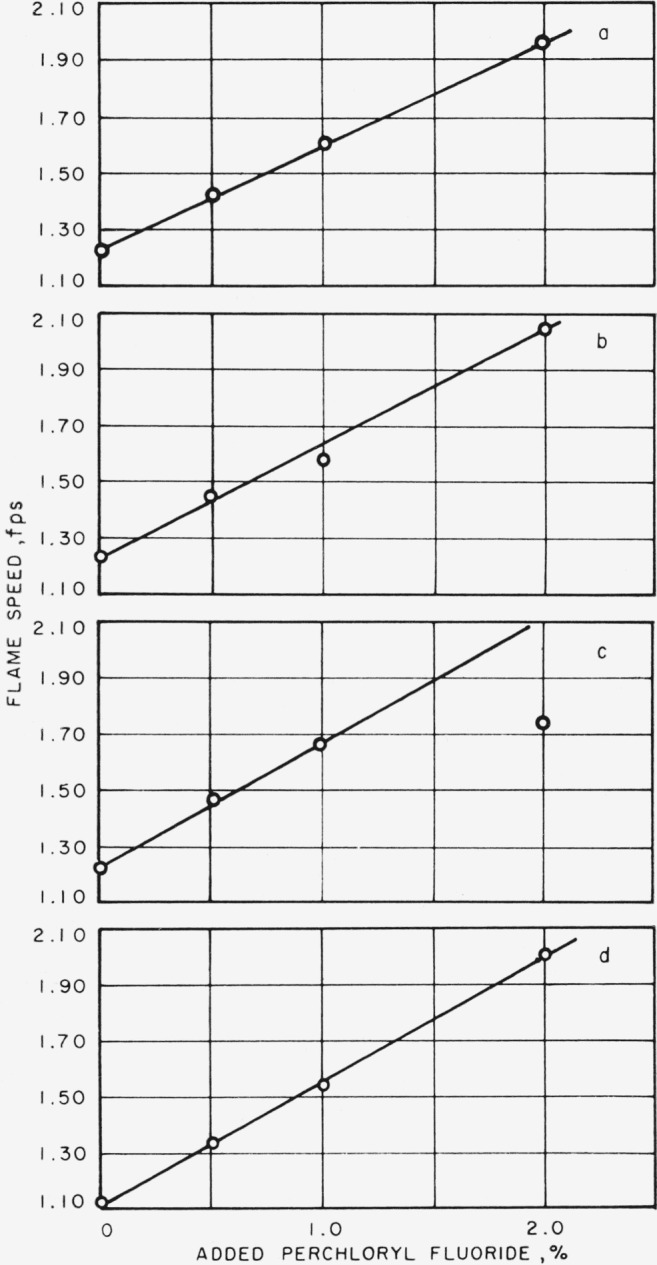
Effect of added perchloryl fluoride on maximum flame speed. Gas velocity=a, 4 fps; b, 5 fps; c, 6 fps; and d, 7 fps.

Results of the addition of perchloryl fluoride are shown in figures 1 and 2. Figure 1 shows the variation of flame speed with mixture ratio, by weight, of methane to air plus perchloryl fluoride at gas velocities, measured at the port of the nozzle, of 4, 5, 6, and 7 fps, when 0.5, 1, 2, and 3 percent by volume, respectively, of perchloryl fluoride was added to the combustion air. Results obtained with burning mixtures made from the 0.5 and 1 percent perchloryl fluoride-air mixtures show the flame speed increasing as the mixture becomes richer in fuel, rising to a maximum and then decreasing. With the 2 percent mixture, the flame speed increases as the burning mixture becomes richer in fuel, and a maximum value seems to occur at mixture ratio of 0.072. Only one set of observations could be made with the 3 percent mixture; at a gas velocity of 6 fps, flame speed increased as the burning mixture became richer in fuel until flashback occurred at a mixture ratio of 0.066. Maximum flame speeds and the experimental conditions at which they occurred are listed in table 2. As may be noted from the data listed in table 2, the addition of perchloryl fluoride to the combustible mixture shifted the mixture ratio at which maximum flame speed occurs. It had been found previously [7] that maximum flame speed, 1.233 fps, for methane-air at a temperature of 84.4 °F occurred at mixture ratio of 0.062. The addition of 2 percent by volume of perchloryl fluoride moved the maximum flame speed to a mixture ratio for methane to air plus perchloryl fluoride of 0.072.

Theoretically, flame speed is a property of the fuel and oxidant and of the physical condition of the mixture, and is independent of the apparatus in which it is measured. Hence, it was not expected that there be any variation of flame speed with gas velocity. However, there proved to be some variation. The 0.5 percent mixture of perchloryl fluoride in air showed practically no variation for lean burning mixtures. At richer mixtures, fuel-air plus perchloryl fluoride ratios of 0.068 and higher, there was a slight decrease of flame speed as gas velocity increases, which amounted to about 1.5 percent of the average value of the flame speed. The 1 percent mixture showed a small increase of flame speed as the gas velocity increases, also with a variation of about 1.5 percent. The 2 percent mixture showed a larger ncrease of flame speed with gas velocity, with a variation of 5 percent. (If the value of flame speed at 6 fps, which seems much too low, is ignored, then the variation becomes about 3 percent.) The flame speeds considered here have been corrected for temperature. The reasons for the variation of flame speed with gas velocity are obscure. Since these variations are small, they may be due to experimental difficulties, such as locating the flame front in the enlargements of the flame photographs, and thus they may fall within the experimental error. However, the trends seem to be consistent and, since a similar variation of flame speed with gas velocity had been found for methane-air flames previously [7], it is felt that the variation does exist. Conditions of the gas flow in the throat of the nozzle, e.g., the presence or absence, and the extent, of a boundary layer, are the most likely cause.

From the data which are listed in table 5, it may be shown that flame speed increases linearly with increasing percentage of perchloryl fluoride, at least over the small range of perchloryl fluoride addition covered here, and as the burning mixture becomes richer in fuel, the rate of increase of flame speed becomes larger. The values of flame speed at 0 percent perchloryl fluoride, that is, for a methane-air flame, are taken from our previous work [7]. In figure 2 is shown the relation of maximum flame speed for each gas velocity to the percentage of perchloryl fluoride added. Maximum flame speed varies linearly with the percentage of added perchloryl fluoride, and the rate of increase at each gas velocity is about the same.

**Figure 3(d):**
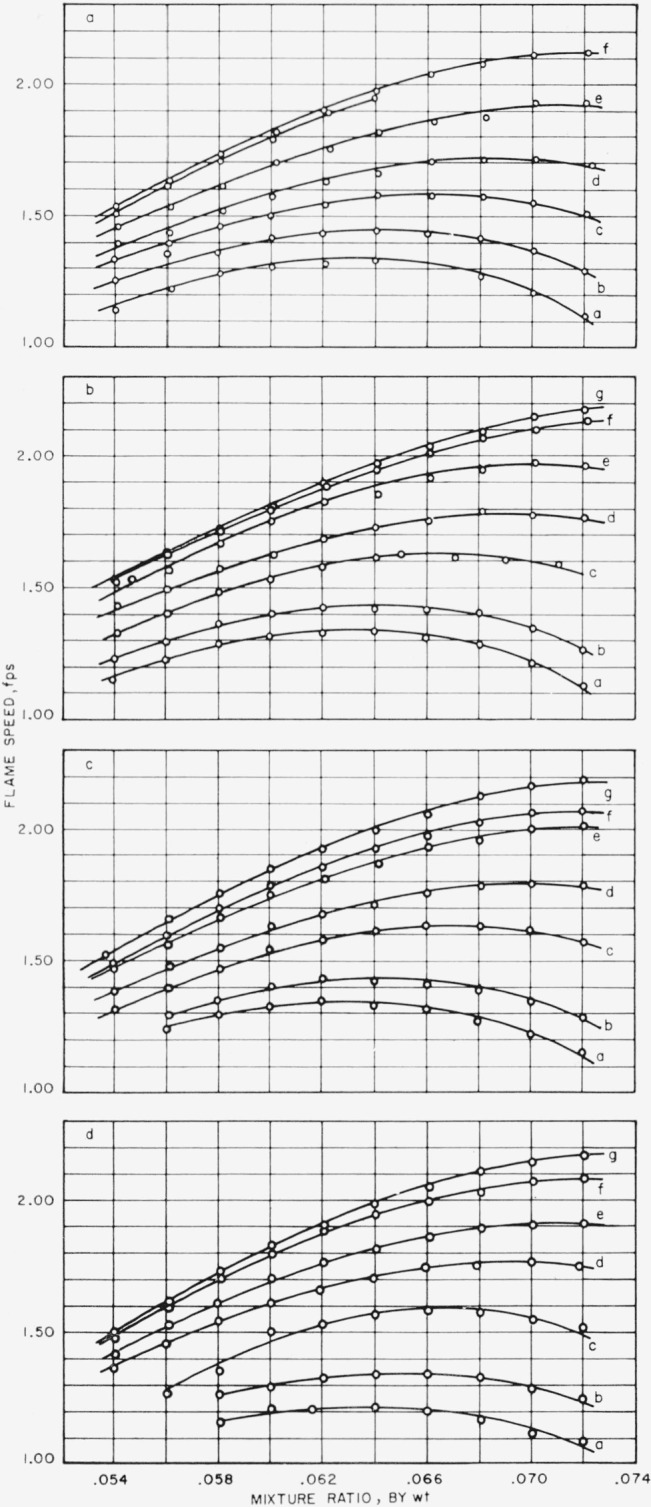
Effect of mixture ratio, by weight, of methane to air plus added oxygen on flame speed. Percent added oxygen=a, 0.5; b, 1.0; c, 2.0; d, 3.0; e, 4.0; f, 5.0; and g, 6.0. Gas velocity=A, 4 fps; B, 5 fps· C, 6 fps; and D, 7 fps.

**Figure 4:**
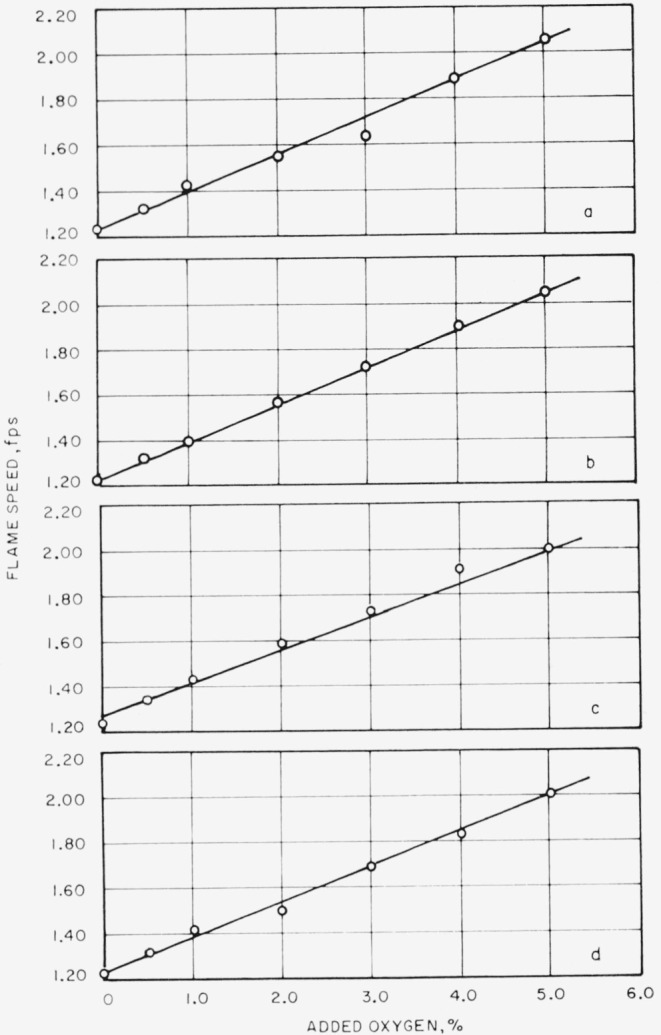
Effect of added oxygen on maximum flame speed. Gas velocity=a, 4 fps; b, 5 fps; c, 6 fps; and d, 7 fps.

Figures 3 and 4 show the effect of addition of oxygen on the flame speed of methane-air mixtures. Figure 3 shows the variation of flame speed with mixture ratio, by weight, of methane to air plus oxygen at constant gas velocity and at various strengths of added oxygen. The maximum flame speeds observed for each set of experimental conditions are listed in table 3. It can be noted in figure 3 that, at low percentages of added oxygen, as the burning mixture becomes richer in fuel, the flame speed increases to a maximum and then decreases. As the burning mixture becomes richer in oxygen, the value of the mixture ratio at which the flame speed is a maximum gradually changes toward the fuel-rich side, and at 5 percent added oxygen, the maximum flame speed occurs about fuel to air plus oxygen ratio of 0.072. With 6 percent added oxygen, the maximum flame speed occurs beyond 0.072; i.e., an inflection point is not observed.

Flame speeds of methane-air mixtures to which oxygen had been added also proved to vary somewhat with gas velocity. In general, the flame speed decreased as the gas velocity increased. However, there were a few instances where an increase of flame speed with increasing gas velocity occurred: at 0.5 percent oxygen addition, at mixture ratio of 0.072; at 1 percent oxygen addition, at mixture ratio of 0.072; and at 6 percent oxygen addition, at mixture ratios of 0.056, 0.058, 0.060, 0.062, 0.064, 0.066, and 0.068. These variations of flame speeds, whether increase or decrease, are small and amount to about 1.2 percent of the average value of the flame speed. The flame speeds considered here have been corrected for temperature.

The data which are displayed in table 6 show the effect of increasing the amount of oxygen in the combustible mixture on the flame speed. It may be shown that flame speed increases linearly as the amount of oxygen is increased up to the addition of 4 or 5 percent; then the rate of increases changes abruptly and becomes less; the increase appears to be still linear. This effect is seen at all mixture ratios of fuel to air plus oxygen that were used and at all gas velocities. However, at gas velocities of 6 and 7 fps, the effect is less pronounced than at 4 and 5 fps. The rate of increase of flame speed (up to 4 or 5 percent oxygen added) is greater with mixtures that are fuel-rich. If, as in figure 4, the maximum flame speed observed for each experimental condition is plotted against the percentage of oxygen added to the combustible mixture, then the result is a straight line and the slopes of the lines at each gas velocity are of similar magnitude. The values of flame speeds for 0 percent oxygen addition, that is, for methane-air, are taken from [7]. Since the maximum addition of oxygen was only 6 percent, one might question if there would not be a break in the curve at large oxygen addition. Lewis and von Elbe [8] reproduce a figure taken from the work of Jahn in which flame speed is plotted against the concentration of methane for “combustion airs” containing from 21 to 98.5 percent by volume of oxygen. A line drawn through the maximum flame speed found for each condition of “combustion air” is practically a straight line, showing a very slight degree of curvature, so that from 21 to 40 percent, by volume, of oxygen, the line is straight. In our experiments oxygen in the “combustion air” varied from 21 to 25.5 percent by volume.

If it is assumed that the relation between maximum flame speed and oxygen content is, in fact, linear, then from the slope of the curves in figure 4 the maximum flame speed of methane in oxygen is estimated to be 16.5 fps (503 cm/sec). For comparison with experimental values, Jahn [9] gives 330 cm/sec, Fiock [10] lists 393 cm/sec, Singer and Heimel [11] give 445 cm/sec, and Singer, Grumer, and Cook [12] give 375 and 440 cm/sec for different types of burners. In a like manner from data given in figure 2, it is possible to estimate the maximum flame speed for methane and perchloryl fluoride to be about 42 fps (1,280 cm/sec). However, since the extrapolation involved is quite large, and the assumed linearity of the relation of maximum flame speed with added perchoryl fluoride over the entire range up to 100 percent perchlorly fluoride quite uncertain, this value of 42 fps should be regarded with caution.

The addition of perchloryl fluoride is more effective than the addition of oxygen in increasing the flame speeds of methane-air mixtures. The greatest flame speed observed in these experiments was 2.386 fps; this occurred with the addition of 3 percent perchloryl fluoride by volume to the combustion air at a mixture ratio, by weight, of methane to air plus perchloryl fluoride of 0.064. The gas velocity was 6 fps, the gas temperature, was 82.0 °F and the water content was 0.03 percent by volume. The greatest flame speed observed on addition of oxygen was 2.187 fps; this was found with the addition of 6 percent oxygen by volume to the combustion air. Mixture ratio, by weight, of methane to air plus oxygen was 0.072, gas velocity was 6 fps, gas temperature was 95.8 °F, and water content was 0.03 percent by volume. The above values for flame speeds should be compared with a maximum flame speed of 1.233 fps for a methane-air flame, mixture ratio, by weight, of methane to air of 0.062, gas velocity of 6 fps, gas temperature of 84.4 °F, and water content, 0.03 percent by volume.

The heat of combustion of methane burning in perchloryl fluoride is calculated to be −215.1 kcal/mole, while the heat of combustion of methane in oxygen is only −191.8 kcal/mole. Since there is more energy available in the perchloryl fluoride reaction, it is to be expected that flame speeds should be larger.

The flame speed of methane-air mixtures had been found previously [7] to depend strongly on the initial temperature of the combustible mixture. Increasing the temperature increased the flame speed. At 330 °F, the highest temperature used, maximum flame speed was 2.463 fps at a mixture ratio, by weight, of methane to air of 0.062. Gas velocity was 5 fps at the port of the nozzle and the water content was 0.03 percent by volume. This value of flame speed should be compared to 2.386 fps, the greatest found for perchloryl fluoride addition, and 2.187 fps for oxygen addition in the ranges covered in this report. From our previous work [7], it was found that to attain a flame speed of 2.386 fps in a methane-air flame, it was necessary to preheat the mixture to about 320 °F and to attain 2.187 fps, an initial gas temperature of about 295 °F is needed. Hence, it is apparent that a moderate increase in the initial temperature of the methane-air mixture is more effective in increasing flame speeds than is the addition of small quantities of other oxidants.

## 4. Experimental Observations

Tables 4, 5, and 6 present in detail some observations on the effect of some variables on the flame speed.

## 5. Conclusions

Both perchloryl fluoride and oxygen increase the flame speed when added to burning mixtures of methane and air, and the effect of perchloryl fluoride, on a molar basis, is greater than that of oxygen. However, preheating the methane-air mixture moderately, up to temperatures of 330° F, is more effective in increasing flame speeds than the addition of small amounts of either oxidant. The mixture ratio at which maximum flame speed occurs is displaced beyond 0.072 by only small additions of either perchloryl fluoride or oxygen. In the range of addition of oxidants covered in this report, the maximum flame speed increases linearly with the addition of either perchloryl fluoride or oxygen, although at different rates.

## Figures and Tables

**Table 1:** Limits of operation of burner

Percent ClO_3_F by volume added to air	Percent O_2_ by volume added to air	$\frac{\text{wt methane}}{\text{wt air}+\text{oxidant}}$	Gas velocity fps

Blowoff			

0.5	0	0.054	6
.5	0	.056	6
.5	0	.054	7
.5	0	.056	7
0	.5	.054	6
0	.5	.054	7
0	.5	.056	7
0	1.0	.054	6
0	1.0	.054	7
0	2.0	.054	7

Flashback			

3.0	0	.066	4
0	6.0	.066	4

**Table 2:** Maximum flame speeds for perchloryl fluoride addition and the experimental conditions at which they were obtained

Flame speed fps	$\frac{\text{wt methane}}{\text{wt air}+{\text{ClO}}_{3}F}$	Percent ClO_3_F by volume added to air	Gas velocity fps	Gas temp. °F
				
1.449	0.068	0.5	4	87.8
1.475	.066	.5	5	88.0
1.480	.066	.5	6	88.1
1.455	.066	.5	7	88.4
1.652	.070	1.0	4	93.1
1.611	.066	1.0	5	90.6
1.705	.070	1.0	6	92.4
1.690	.072	1.0	7	94.4
1.970	.072	2.0	4	85.7
2.082	.072	2.0	5	90.3
1.770	.072	2.0	6	89.7
2.123	.072	2.0	7	88.4
2.386	.064*	3.0	6	82.0

* Flashback occurred at 0.066.

**Table 3:** Maximum flame speeds for oxygen addition and the experimental conditions at which they were obtained

Flame speed fps	$\frac{\text{wt methane}}{\text{wt air}+\text{oxygen}}$	Percent O_2_ by volume added to air	Gas velocity fps	Gas temp. °F
				
1.322	0.064	0.5	4	84.9
1.333	.064	.5	5	84.3
1.343	.062	.5	6	87.2
1.318	.064	.5	7	86.9
1.440	.064	1.0	4	88.2
1.422	.062	1.0	5	88.6
1.436	.062	1.0	6	88.0
1.443	.066	1.0	7	91.7
1.583	.064	2.0	4	92.4
1.634	.065	2.0	5	95.7
1.634	.068	2.0	6	97.4
1.581	.066	2.0	7	95.2
1.707	.070	3.0	4	100.0
1.788	.068	3.0	5	97.0
1.786	.070	3.0	6	98.4
1.765	.070	3.0	7	101.3
1.930	.070	4.0	4	95.3
1.970	.072	4.0	5	98.4
2.004	.072	4.0	6	102.4
1.915	.072	4.0	7	102.4
2.118	.072	5.0	4	98.3
2.139	.072	5.0	5	100.5
2.067	.072	5.0	6	101.2
2.080	.072	5.0	7	101.9
1.943	*.064	6.0	4	91.2
2.169	> .072	6.0	5	93.8
2.187	> .072	6.0	6	95.8
2.172	> .072	6.0	7	93.9

* Flash-back occurred at 0.066.

**Table 4:** Effect of some variables on flame speed

$\frac{\text{Wt methane}}{\text{Wt air}+{\text{ClO}}_{3}F}$	Flame speed fps	Gas temperature °F	$\frac{\text{Wt methane}}{\text{Wt air}+C_{2}}$	Flame speed fps	Gas temperature °F

A. 0.5 percent by volume ClO_3_F added to air					

1. Gas velocity=4 fps			2. Gas velocity=5 fps		
0.05404	1.172	85.2	0.05407	1.159	88.5
.05597	1.233	85.7	.05661	1.241	85.2
.05805	1.303	86.1	.05809	1.315	86.1
.06017	1.320	86.4	.06003	1.385	86.7
.06217	1.376	86.8	.06212	1.418	87.2
.06414	1.418	87.2	.06406	1.470	87.8
.06603	1.445	87.4	.06606	1.475	88.0
.06802	1.449	87.8	.06806	1.462	88.4
.07006	1.421	88.0	.07014	1.429	88.9
.07191	1.400	87.4	.07212	1.412	88.9

3. Gas velocity=6 fps			4. Gas velocity=7 fps		
0.05802	1.315	88.6	0.05804	1.320	89.3
.06003	1.379	85.7	.06002	1.379	89.7
.06206	1.430	86.7	.06200	1.404	90.1
.06401	1.468	87.3	.06404	1.406	88.3
.06601	1.480	88.1	.06608	1.455	88.4
.06804	1.458	88.6	.06797	1.434	89.0
.07002	1.414	89.2	.07006	1.391	89.4
.07201	1.384	89.6	.07201	1.370	89.9

B. 1 percent volume ClO_3_F added to air					

1. Gas velocity=4 fps			2. Gas velocity=5 fps		
0.05404	1.250	87.9	0.05496	1.234	91.0
.05603	1.328	88.6	.05701	1.321	91.0
.05802	1.380	89.2	.05903	1.406	90.6
.06012	1.504	89.8	.06108	1.473	91.8
.06201	1.578	90.3	.06312	1.546	89.9
.06410	1.600	90.7	.06513	1.580	90.6
.06603	1.641	91.2	.06669	1.611	91.1
.06807	1.646	92.8	.06926	1.608	89.6
.07007	1.652	93.0	.07128	1.600	89.6
.07208	1.637	93.1	. 07332	1.587	91.6

3. Gas velocity=6 fps			4. Gas velocity=7 fps		
0.05404	1.306	91.1	0.05606	1.403	95.1
.05605	1.401	91.5	.05815	1.452	91.9
.05780	1.457	91.4	.06027	1.510	91.4
.06015	1.526	90.6	.06208	1.569	88.2
.06204	1.627	93.4	.06411	1.625	90.0
.06402	1.646	91.5	.06609	1.664	91.2
.06606	1.674	90.8	.06735	1.676	92.7
.06802	1.690	91.8	.07011	1.690	94.4
.07003	1.705	92.4	.07205	1.669	95.6
.07204	1.690	93.2			

C. 2 percent by volume ClO_3_F added to air					

1. Gas velocity=4 fps			2. Gas velocity=5 fps		
0.05403	1.381	82.2	0.05402	1.435	84.9
.05596	1.482	82.5	.05601	1.534	86.0
.05798	1.544	83.1	.05807	1.681	86.5
.06003	1.649	83.6	.06004	1.712	86.6
.06197	1.719	83.6	.06207	1.828	87.7
.06404	1.754	85.0	.06407	1.869	88.4
.06597	1.871	85.0	.06604	1.969	88.9
.06801	1.903	85.6	.06809	1.987	89.0
.07000	1.945	85.9	.07009	2.043	89.7
.07200	1.970	85.7	.07201	2.082	90.3
	
3. Gas velocity=6 fps			4. Gas velocity=7 fps		
0.05409	1.357	87.9	0.05397	1.527	83.4
.05609	1.474	88.4	.05600	1.632	84.2
.05806	1.530	88.8	.05799	1.697	84.8
.06006	1.593	89.8	.06000	1.800	85.2
.06207	1.609	84.4	.06187	1.889	85.8
.06404	1.660	86.4	.06401	1.969	86.0
.06597	1.710	87.8	.06600	2.027	86.5
.06801	1.743	88.4	.06796	2.036	87.2
.07002	1.758	89.1	.06999	2.092	87.9
.07206	1.770	89.7	.07199	2.123	88.4

$\frac{\text{Wt methane}}{\text{Wt air}+{\text{ClO}}_{3}F}$	Flame speed fps	Gas temperature °F	$\frac{\text{Wt methane}}{\text{Wt air}+O_{2}}$	Flame speed fps	Gas temperature °F

D. 3 percent by volume ClO_3_F added to air			A. 0.5 percent by volume O_2_ added to air		
	
1. Gas velocity=6 fps			1. Gas velocity=4 fps		
0.05400	1.771	80.0	0.05411	1.140	80.4
.05601	1.904	79.8	.05612	1.223	81.4
.05802	2.079	80.4	.05807	1.279	82.4
.06003	2.168	80.3	.06031	1.305	83.4
.06205	2.276	80.6	.06210	1.318	84.3
.06400	2.386	82.0	.06399	1.322	84.9
			.06807	1.268	86.0
			.07001	1.209	86.4
			.07210	1.129	86.8

$\frac{\text{Wt methane}}{\text{Wt air}+O_{2}}$	Flame speed fps	Gas temperature °F	$\frac{\text{Wt methane}}{\text{Wt air}+O_{2}}$	Flame speed fps	Gas temperature °F

A. 0.5 percent by volume O_2_ added to air					

2. Gas velocity=5 fps			3. Gas velocity=6 fps		
0.05398	1.147	81.3	0.05600	1.240	86.1
.05598	1.221	81.7	.05803	1.297	86.6
.05802	1.280	82.5	.06000	1.326	86.9
.05999	1.312	83.1	.06200	1.343	87.2
.06201	1.327	83.8	.06399	1.329	84.0
.06401	1.333	84.3	.06600	1.319	84.8
.06598	1.308	85.1	.06799	1.268	85.7
.06799	1.284	85.6	.07001	1.223	86.4
.07003	1.215	86.1	.07199	1.158	86.9
.07203	1.127	86.6			
	
A. 0.5 percent by volume O_2_ added to air			B. 1.0 percent by volume O_2_ added to air		
	
4. Gas velocity=7 fps			1. Gas velocity=4 fps		
0.05803	1.258	89.7	0.05404	1.255	84.3
.06005	1.305	90.5	.05599	1.358	85.3
.06172	1.305	86.3	.05796	1.359	86.1
.06400	1.318	86.9	.06004	1.418	86.7
.06601	1.301	87.6	.06202	1.432	87.4
06804	1.271	88.5	.06406	1.440	88.2
.07001	1.219	89.4	.06596	1.433	88.0
.07201	1.187	90.4	.06801	1.416	89.2
			.07003	1.363	89.7
			.07206	1.298	90.1

B. 1.0 percent by volume O_2_ added to air					

2. Gas velocity=5 fps			3. Gas velocity=6 fps		
0.05406	1.224	86.0	0.05604	1.293	90.6
.05598	1.284	86.8	.05799	1.352	90.8
.05801	1.359	87.4	.06003	1.404	91.3
.06003	1.400	88.0	.06203	1.436	88.0
.06201	1.422	88.6	.06400	1.426	88.8
.06402	1.418	89.4	.06600	1.411	89.4
.06607	1.417	89.9	.06802	1.384	90.1
.06803	1.402	90.3	.07003	1.347	90.7
.07006	1.342	90.6	.07202	1.290	91.1
.07204	1.267	91.0			
	
B. 1.0 percent by volume O_2_ added to air			C. 2.0 percent by volume O_2_ added to air		
	
4. Gas velocity=7 fps			1. Gas velocity=4 fps		
0.05802	1.365	93.6	0.05403	1.333	93.6
.05997	1.388	87.4	.05607	1.388	89.6
.06202	1.422	88.7	.05807	1.459	90.4
.06401	1.440	89.8	.05999	1.497	91.0
.06598	1.443	91.7	.06207	1.538	91.7
.06803	1.429	92.4	.06404	1.583	92.4
.07000	1.389	92.8	.06612	1.579	92.8
.07196	1.351	93.7	.06813	1.571	93.3
			.07008	1.543	93.7
			.07216	1.507	94.0

2. Gas velocity=5 fps			3. Gas velocity=6 fps		
0.05412	1.334	97.0	0.05405	1.319	97.3
.05604	1.400	92.4	.05609	1.397	93.2
.05804	1.480	93.4	.05808	1.467	94.1
.06008	1.531	94.0	.06004	1.540	94.7
.06206	1.579	94.5	.06206	1.574	95.4
.06308	1.611	95.1	.06407	1.614	96.3
.06502	1.634	95.7	.06602	1.622	96.7
.06704	1.617	96.1	.06804	1.634	97.4
.06898	1.607	96.7	.06999	1.613	97.8
.07104	1.583	97.1	.07205	1.576	98.4

C. 2.0 percent by volume O_2_ added to air			D. 3.0 percent by volume of O_2_ added to air		
	
4. Gas velocity=7 fps			1. Gas velocity=4 fps		
0.05602	1.371	96.6	0.05411	1.388	99.0
.05802	1.452	95.3	.05606	1.433	94.2
.06001	1.506	93.3	.05811	1.516	95.4
.06203	1.536	93.9	.06004	1.571	96.4
.06405	1.574	94.7	.06213	1.626	97.4
.06602	1.581	95.2	.06409	1.659	98.1
.06805	1.579	96.0	.06615	1.704	98.8
.07001	1.551	96.5	.06815	1.707	99.4
.07200	1.523	97.3	.07011	1.707	100.0
			.07226	1.696	100.5

D. 3.0 percent by volume of O_2_ added to air			3. Gas velocity=6 fps		
2. Gas velocity=5 fps					
	
0.05413	1.429	97.7	0.05408	1.386	98.4
.05608	1.491	99.3	.05610	1.479	93.7
.05809	1.563	94.4	.05808	1.554	94.8
.06010	1.624	95.0	.06006	1.627	95.5
.06210	1.679	95.4	.06204	1.672	96.0
.06407	1.724	96.1	.06404	1.714	96.8
.06610	1.758	96.7	.06608	1.754	97.4
.06813	1.788	97.0	.06806	1.784	98.0
.07011	1.776	97.7	.07007	1.786	98.4
.07208	1.767	98.0	.07204	1.781	98.7

D. 3.0 percent by volume O_2_ added to air			E. 4.0 percent by volume O_2_ added to air		
	
4. Gas velocity=7 fps			1. Gas velocity=4 fps		
0.05396	1.359	100.4	0.05415	1.459	91.0
.05594	1.454	100.7	.05617	1.532	91.4
.05797	1.540	101.1	.05816	1.612	92.0
.05992	1.612	101.9	.06020	1.692	92.6
.06189	1.660	96.9	.06222	1.752	93.2
.06391	1.704	98.2	.06415	1.818	93.7
.06593	1.749	99.4	.06624	1.851	94.2
.06793	1.756	100.5	.06822	1.866	94.5
.06995	1.765	101.3	.07018	1.930	94.9
.07188	1.756	102.3	.07226	1.924	95.3

2. Gas velocity=5 fps			3. Gas velocity=6 fps		
0.05467	1.522	93.1	0.05405	1.476	95.4
.05609	1.571	93.8	.05607	1.559	96.3
.05806	1.668	94.5	.05810	1.664	97.3
.06008	1.746	95.2	.06004	1.749	98.4
.06210	1.822	95.7	.06209	1.809	99.1
.06416	1.853	96.6	.06414	1.864	99.8
.06612	1.915	97.1	.06607	1.932	100.4
.06811	1.950	97.8	.06807	1.959	101.1
.07012	1.974	98.4	.07006	1.996	101.8
.07210	1.970	99.0	.07202	2.004	102.4

E. 4.0 percent by volume O_2_ added to air			F. 5.0 percent by volume O_2_ added to air		
	
4. Gas velocity=7 fps			1. Gas velocity=4 fps		
0.05403	1.418	102.2	0.05406	1.536	93.2
.05604	1.526	100.8	.05608	1.632	94.3
.05797	1.604	100.7	.05805	1.729	94.9
.06002	1.702	100.8	.06016	1.815	95.4
.06205	1.763	101.3	.06216	1.894	96.0
.06404	1.816	101.9	.06403	1.970	96.7
.06602	1.872	102.3	.06610	2.035	97.1
.06804	1.897	102.7	.06811	2.073	97.6
.07003	1.908	102.8	.07014	2.108	97.8
.07197	1.915	102.0	.07216	2.118	98.3

F. 5.0 percent by volume O_2_ added to air					

2. Gas velocity=5 fps			3. Gas velocity=6 fps		
0.05405	1.516	94.3	0.05400	1.487	94.0
.05606	1.624	95.3	.05608	1.593	95.3
.05805	1.714	96.2	.05808	1.697	96.5
.06008	1.794	97.0	.06004	1.786	97.3
.06214	1.883	97.7	.06203	1.849	98.0
.06409	1.949	98.1	.06408	1.926	98.6
.06610	2.004	98.7	.06605	1.970	99.3
.06814	2.068	99.4	.06805	2.025	100.0
.07013	2.097	99.9	.07006	2.063	100.5
.07217	2.139	100.5	.07204	2.067	101.2

F. 5.0 percent by volume O_2_ added to air			G. 6.0 percent by volume O_2_ added to air		
	
4. Gas velocity=7 fps			1. Gas velocity=4 fps		
0.05400	1.478	92.8	0.05403	1.501	87.2
.05603	3.594	95.0	.05603	1.610	88.2
.05802	1.708	96.4	.05808	1.704	88.9
.06002	1.796	97.7	.06006	1.791	89.9
.06202	1.887	98.6	.06206	1.898	90.6
.06401	1.950	99.3	.06402	1.943	91.2
.06602	1.996	99.9			
.06802	2.036	100.6			
.07005	2.068	101.3			
.07202	2.080	101.9			

G. 6.0 percent by volume O_2_ added to air					

2. Gas velocity=5 fps			3. Gas velocity=6 fps		
0.05404	1.514	88.1	0.05366	1.526	90.1
.05608	1.620	89.0	.05605	1.660	90.5
.05802	1.719	89.7	.05808	1.755	91.0
.06009	1.800	90.3	.06004	1.846	91.8
.06205	1.884	90.8	.06208	1.930	92.6
.06407	1.963	91.5	.06404	2.001	93.1
.06607	2.029	92.1	.06604	2.062	94.0
.06812	2.090	92.8	.06806	2.126	94.4
.07011	2.144	93.3	.07006	2.169	95.1
.07208	2.169	93.8	.07203	2.187	95.8

4. Gas velocity=7 fps					

0.05403	1.494	88.0	.06396	1.986	91.4
.05605	1.610	88.7	.06604	2.054	92.0
.05807	1.734	89.5	.06802	2.111	92.7
.06001	1.833	90.2	.07002	2.146	93.4
.06202	1.904	90.9	.07201	2.172	93.9

**Table 5:** Effect of perchloryl fluoride addition on flame speed of methane

Vol. percent ClO_3_F added to air	Flame speed fps	Vol. percent ClO_3_F added to air	Flame speed fps

A. Gas velocity=4 fps			

$1.\frac{\text{Wt methane}}{\text{Wt air}+{\text{ClO}}_{3}F}=0.054$		$2.\frac{\text{Wt methane}}{\text{Wt air}+{\text{ClO}}_{3}F}=0.056$	
	
0.0	1.094	0.0	1.156
0.5	1.168	0.5	1.226
1.0	1.232	1.0	1.304
2.0	1.392	2.0	1.492

$3.\frac{\text{Wt methane}}{\text{Wt air}+{\text{ClO}}_{3}F}=0.058$		$4.\frac{\text{Wt methane}}{\text{Wt air}+{\text{ClO}}_{3}F}=0.060$	
	
0.0	1.188	0.0	1.219
0.5	1.294	0.5	1.310
1.0	1.356	1.0	1.477
2.0	1.548	2.0	1.653

$5.\frac{\text{Wt methane}}{\text{Wt air}+{\text{ClO}}_{3}F}=0.062$		$6.\frac{\text{Wt methane}}{\text{Wt air}+{\text{ClO}}_{3}F}=0.064$	
	
0.0	1.233	0.0	1.223
0.5	1.364	0.5	1.404
1.0	1.548	1.0	1.568
2.0	1.723	2.0	1.751

$7.\frac{\text{Wt methane}}{\text{Wt air}+{\text{ClO}}_{3}F}=0.066$		$8.\frac{\text{Wt methane}}{\text{Wt air}+{\text{ClO}}_{3}F}=0.068$	
	
0.0	1.190	0.0	1.148
0.5	1.433	0.5	1.432
1.0	1.622	1.0	1.604
2.0	1.868	2.0	1.897

$9.\frac{\text{Wt methane}}{\text{Wt air}+{\text{ClO}}_{3}F}=0.070$		$10.\frac{\text{Wt methane}}{\text{Wt air}+{\text{ClO}}_{3}F}=0.072$	
	
0.0	1.094	0.0	1.017
0.5	1.403	0.5	1.385
1.0	1.609	1.0	1.593
2.0	1.937	2.0	1.963

B. Gas velocity=5 fps			

$1.\frac{\text{Wt methane}}{\text{Wt air}+{\text{ClO}}_{3}F}=0.054$		$2.\frac{\text{Wt methane}}{\text{Wt air}+{\text{ClO}}_{3}F}=0.056$	
	
0.0	1.094	0.0	1.156
0.5	1.138	0.5	1.237
1.0	1.201	1.0	1.288
2.0	1.432	2.0	1.526

$3.\frac{\text{Wt methane}}{\text{Wt air}+{\text{ClO}}_{3}F}=0.058$		$4.\frac{\text{Wt methane}}{\text{Wt air}+{\text{ClO}}_{3}F}=0.060$	
	
0.0	1.188	0.0	1.219
0.5	1.306	0.5	1.372
1.0	1.375	1.0	1.436
2.0	1.436	2.0	1.670

$5.\frac{\text{Wt methane}}{\text{Wt air}+{\text{ClO}}_{3}F}=0.062$		$6.\frac{\text{Wt methane}}{\text{Wt air}+{\text{ClO}}_{3}F}=0.064$	
	
0.0	1.233	0.0	1.223
0.5	1.404	0.5	1.453
1.0	1.518	1.0	1.549
2.0	1.701	2.0	1.849

$7.\frac{\text{Wt methane}}{\text{Wt air}+{\text{ClO}}_{3}F}=0.066$		$8.\frac{\text{Wt methane}}{\text{Wt air}+{\text{ClO}}_{3}F}=0.068$	
	
0.0	1.190	0.0	1.148
0.5	1.457	0.5	1.442
1.0	1.577	1.0	1.582
2.0	1.946	2.0	1.964

$9.\frac{\text{Wt methane}}{\text{Wt air}+{\text{ClO}}_{3}F}=0.070$		$10.\frac{\text{Wt methane}}{\text{Wt air}+{\text{ClO}}_{3}F}=0.072$	
	
0.0	1.094	0.0	1.017
0.5	1.406	0.5	1.389
1.0	1.574	1.0	1.551
2.0	2.016	2.0	2.052

C. Gas velocity=6 fps			

$1.\frac{\text{Wt methane}}{\text{Wt air}+{\text{ClO}}_{3}F}=0.054$		$2.\frac{\text{Wt methane}}{\text{Wt air}+{\text{ClO}}_{3}F}=0.056$	
	
0.0	1.094	0.0	1.156
0.5	………….	0.5	…………….
1.0	1.272	1.0	1.365
2.0	1.339	2.0	1.452
3.0	1.749	3.0	1.881

$3.\frac{\text{Wt methane}}{\text{Wt air}+{\text{ClO}}_{3}F}=0.058$		$4.\frac{\text{Wt methane}}{\text{Wt air}+{\text{ClO}}_{3}F}=0.060$	
	
0.0	1.188	0.0	1.219
0.5	1.304	0.5	1.372
1.0	1.422	1.0	1.495
2.0	1.508	2.0	1.566
3.0	2.059	3.0	2.147

$5.\frac{\text{Wt methane}}{\text{Wt air}+{\text{ClO}}_{3}F}=0.062$		$6.\frac{\text{Wt methane}}{\text{Wt air}+{\text{ClO}}_{3}F}=0.064$	
	
0.0	1.233	0.0	1.223
0.5	1.418	0.5	1.453
1.0	1.582	1.0	1.610
2.0	1.589	2.0	1.650
3.0	2.257	3.0	2.374

$7.\frac{\text{Wt methane}}{\text{Wt air}+{\text{ClO}}_{3}F}=0.066$		$8.\frac{\text{Wt methane}}{\text{Wt air}+{\text{ClO}}_{3}F}=0.068$	
	
0.0	1.190	0.0	1.148
0.5	1.461	0.5	1.437
1.0	1.632	1.0	1.653
2.0	1.693	2.0	1.723

$9.\frac{\text{Wt methane}}{\text{Wt air}+{\text{ClO}}_{3}F}=0.070$		$10.\frac{\text{Wt methane}}{\text{Wt air}+{\text{ClO}}_{3}F}=0.072$	
	
0.0	1.094	0.0	1.017
0.5	1.390	0.5	1.358
1.0	1.665	1.0	1.646
2.0	1.734	2.0	1.743

D. Gas velocity=7 fps			

$1.\frac{\text{Wt methane}}{\text{Wt air}+{\text{ClO}}_{3}F}=0.054$		$2.\frac{\text{Wt methane}}{\text{Wt air}+{\text{ClO}}_{3}F}=0.056$	
	
0.0	1.094	0.0	1.156
0.5	…..	0.5	…..
1.0	…..	1.0	1.349
2.0	1.532	2.0	1.633

$3.\frac{\text{Wt methane}}{\text{Wt air}+{\text{ClO}}_{3}F}=0.058$		$4.\frac{\text{Wt methane}}{\text{Wt air}+{\text{ClO}}_{3}F}=0.060$	
	
0.0	1.188	0.0	1.219
0.5	1.295	0.5	1.352
1.0	1.414	1.0	1.475
2.0	1.695	2.0	1.760

$5.\frac{\text{Wt methane}}{\text{Wt air}+{\text{ClO}}_{3}F}=0.062$		$6.\frac{\text{Wt methane}}{\text{Wt air}+{\text{ClO}}_{3}F}=0.064$	
	
0.0	1.233	0.0	1.223
0.5	1.375	0.5	1.386
1.0	1.550	1.0	1.597
2.0	1.882	2.0	1.961

$7.\frac{\text{Wt methane}}{\text{Wt air}+{\text{ClO}}_{3}F}=0.066$		$8.\frac{\text{Wt methane}}{\text{Wt air}+{\text{ClO}}_{3}F}=0.068$	
	
0.0	1.190	0.0	1.148
0.5	1.435	0.5	1.411
1.0	1.630	1.0	1.634
2.0	2.016	2.0	2.022

$9.\frac{\text{Wt methane}}{\text{Wt air}+{\text{ClO}}_{3}F}=0.070$		$10.\frac{\text{Wt methane}}{\text{Wt air}+{\text{ClO}}_{3}F}=0.072$	
	
0.0	1.094	0.0	1.017
0.5	1.366	0.5	1.342
1.0	1.640	1.0	1.613
2.0	2.074	2.0	2.103

**Table 6:** Effect of oxygen addition on flame speed of methane

Vol percent O_2_ added to air	Flame speed fps	Vol percent O_2_ added to air	Flame speed fps

A. Gas velocity =4 fps			

$1.\frac{\text{Wt methane}}{\text{Wt air}+O_{2}}=0.054$		$2.\frac{\text{Wt methane}}{\text{Wt air}+O_{2}}=0.056$	
	
0.0	1.074	0.0	1.079
0.5	1.140	0.5	1.223
1.0	1.233	1.0	1.338
2.0	1.267	2.0	1.347
3.0	1.295	3.0	1.369
4.0	1.406	4.0	1.482
5.0	1.472	5.0	1.567
6.0	1.467	6.0	1.576

$3.\frac{\text{Wt methane}}{\text{Wt air}+O_{2}}=0.058$		$4.\frac{\text{Wt methane}}{\text{Wt air}+O_{2}}=0.060$	
	
0.0	1.178	0.0	1.214
0.5	1.279	0.5	1.305
1.0	1.340	1.0	1.401
2.0	1.419	2.0	1.459
3.0	1.451	3.0	1.506
4.0	1.564	4.0	1.646
5.0	1.666	5.0	1.755
6.0	1.671	6.0	1.758

$5.\frac{\text{Wt methane}}{\text{Wt air}+O_{2}}=0.062$		$6.\frac{\text{Wt methane}}{\text{Wt air}+O_{2}}=0.064$	
	
0.0	1.232	0.0	1.223
0.5	1.318	0.5	1.322
1.0	1.416	1.0	1.421
2.0	1.501	2.0	1.543
3.0	1.560	3.0	1.590
4.0	1.707	4.0	1.771
5.0	1.825	5.0	1.908
6.0	1.866	6.0	1.909

$7.\frac{\text{Wt methane}}{\text{Wt air}+O_{2}}=0.066$		$8.\frac{\text{Wt methane}}{\text{Wt air}+O_{2}}=0.068$	
	
0.0	1.190	0.0	1.148
0.5	……………	0.5	1.260
1.0	1.411	1.0	1.392
2.0	1.537	2.0	1.472
3.0	1.632	3.0	1.632
4.0	1.802	4.0	1.815
5.0	1.971	5.0	2.007

$9.\frac{\text{Wt methane}}{\text{Wt air}+O_{2}}=0.070$		$10.\frac{\text{Wt methane}}{\text{Wt air}+O_{2}}=0.072$	
	
0.0	1.094	0.0	1.017
0.5	1.200	0.5	1.117
1.0	1.336	1.0	1.269
2.0	1.496	2.0	1.459
3.0	1.629	3.0	1.615
4.0	1.877	4.0	1.871
5.0	2.041	5.0	2.048

B. Gas velocity=5 fps			

$1.\frac{\text{Wt methane}}{\text{Wt air}+O_{2}}=0.054$		$2.\frac{\text{Wt methane}}{\text{Wt air}+O_{2}}=0.056$	
	
0.0	1.078	0.0	1.142
0.5	1.147	0.5	1.221
1.0	1.200	1.0	1.258
2.0	1.255	2.0	1.348
3.0	1.347	3.0	1.409
4.0	1.463	4.0	1.510
5.0	1.451	5.0	1.556
6.0	1.480	6.0	1.583

$3.\frac{\text{Wt methane}}{\text{Wt air}+O_{2}}=0.058$		$4.\frac{\text{Wt methane}}{\text{Wt air}+O_{2}}=0.060$	
	
0.0	1.178	0.0	1.212
0.5	1.280	0.5	1.312
1.0	1.334	1.0	1.375
2.0	1.425	2.0	1.476
3.0	1.503	3.0	1.564
4.0	1.608	4.0	1.685
5.0	1.645	5.0	1.724
6.0	1.688	6.0	1.764

$5.\frac{\text{Wt methane}}{\text{Wt air}+O_{2}}=0.062$		$6.\frac{\text{Wt methane}}{\text{Wt air}+O_{2}}=0.064$	
	
0.0	1.230	0.0	1.222
0.5	1.327	0.5	1.333
1.0	1.398	1.0	1.392
2.0	1.525	2.0	1.557
3.0	1.621	3.0	1.665
4.0	1.762	4.0	1.771
5.0	1.813	5.0	1.908
6.0	1.849	6.0	1.927

$7.\frac{\text{Wt methane}}{\text{Wt air}+O_{2}}=0.066$		$8.\frac{\text{Wt methane}}{\text{Wt air}+O_{2}}=0.068$	
	
0.0	1.190	0.0	1.148
0.5	1.304	0.5	1.278
1.0	1.389	1.0	1.372
2.0	1.577	2.0	1.558
3.0	1.665	3.0	1.725
4.0	1.851	4.0	1.883
5.0	1.932	5.0	1.990
6.0	1.990	6.0	2.048

$9.\frac{\text{Wt methane}}{\text{Wt air}+O_{2}}=0.070$		$10.\frac{\text{Wt methane}}{\text{Wt air}+O_{2}}=0.072$	
	
0.0	1.094	0.0	1.017
0.5	1.206	0.5	1.116
1.0	1.311	1.0	1.234
2.0	1.545	2.0	1.519
3.0	1.710	3.0	1.699
4.0	1.904	4.0	1.897
5.0	2.019	5.0	2.058
6.0	2.099	6.0	2.122

C. Gas velocity=6 fps			

$1.\frac{\text{Wt methane}}{\text{Wt air}+O_{2}}=0.054$		$2.\frac{\text{Wt methane}}{\text{Wt air}+O_{2}}=0.056$	
	
0.0	1.094	0.0	1.156
0.5	…..	0.5	1.231
1.0	…..	1.0	1.262
2.0	1.254	2.0	1.353
3.0	1.316	3.0	1.432
4.0	1.421	4.0	1.499
5.0	1.439	5.0	1.538
6.0	1.497	6.0	1.629

$3.\frac{\text{Wt methane}}{\text{Wt air}+O_{2}}=0.058$		$4.\frac{\text{Wt methane}}{\text{Wt air}+O_{2}}=0.060$	
	
0.0	1.188	0.0	1.219
0.5	1.286	0.5	1.313
1.0	1.320	1.0	1.369
2.0	1.418	2.0	1.488
3.0	1.500	3.0	1.571
4.0	1.599	4.0	1.679
5.0	1.636	5.0	1.721
6.0	1.722	6.0	1.809

$5.\frac{\text{Wt methane}}{\text{Wt air}+O_{2}}=0.062$		$6.\frac{\text{Wt methane}}{\text{Wt air}+O_{2}}=0.064$	
	
0.0	1.233	0.0	1.221
0.5	1.329	0.5	1.329
1.0	1.418	1.0	1.402
2.0	1.519	2.0	1.552
3.0	1.614	3.0	1.650
4.0	1.735	4.0	1.785
5.0	1.781	5.0	1.853
6.0	1.889	6.0	1.955

$7.\frac{\text{Wt methane}}{\text{Wt air}+O_{2}}=0.066$		$8.\frac{\text{Wt methane}}{\text{Wt air}+O_{2}}=0.068$	
	
0.0	1.190	0.0	1.148
0.5	1.317	0.5	1.261
1.0	1.386	1.0	1.355
2.0	1.560	2.0	1.569
3.0	1.689	3.0	1.716
4.0	1.852	4.0	1.875
5.0	1.895	5.0	1.947
6.0	2.014	6.0	2.076

$9.\frac{\text{Wt methane}}{\text{Wt air}+O_{2}}=0.070$		$10.\frac{\text{Wt methane}}{\text{Wt air}+O_{2}}=0.072$	
	
0.0	1.094	0.0	1.017
0.5	1.213	0.5	1.146
1.0	1.315	1.0	1.256
2.0	1.546	2.0	1.506
3.0	1.716	3.0	1.709
4.0	1.909	4.0	1.914
5.0	1.982	5.0	1.983
6.0	2.115	6.0	2.130

D. Gas velocity=7 fps			

$1.\frac{\text{Wt methane}}{\text{Wt air}+O_{2}}=0.054$		$2.\frac{\text{Wt methane}}{\text{Wt air}+O_{2}}=0.056$	
	
0.0	1.094	0.0	1.156
0.5	…..	0.5	…..
1.0	…..	1.0	…..
2.0	…..	2.0	1.310
3.0	1.279	3.0	1.372
4.0	1.329	4.0	1.444
5.0	1.436	5.0	1.541
6.0	1.476	6.0	1.588

$3.\frac{\text{Wt methane}}{\text{Wt air}+O_{2}}=0.058$		$4.\frac{\text{Wt methane}}{\text{Wt air}+O_{2}}=0.060$	
	
0.0	1.188	0.0	1.219
0.5	1.231	0.5	1.274
1.0	1.319	1.0	1.373
2.0	1.397	2.0	1.461
3.0	1.456	3.0	1.524
4.0	1.522	4.0	1.620
5.0	1.648	5.0	1.729
6.0	1.708	6.0	1.804

$5.\frac{\text{Wt methane}}{\text{Wt air}+O_{2}}=0.062$		$6.\frac{\text{Wt methane}}{\text{Wt air}+O_{2}}=0.064$	
	
0.0	1.233	0.0	1.223
0.5	1.295	0.5	1.306
1.0	1.400	1.0	1.413
2.0	1.488	2.0	1.522
3.0	1.561	3.0	1.590
4.0	1.678	4.0	1.728
5.0	1.816	5.0	1.825
6.0	1.871	6.0	1.951

$7.\frac{\text{Wt methane}}{\text{Wt air}+O_{2}}=0.066$		$8.\frac{\text{Wt methane}}{\text{Wt air}+O_{2}}=0.068$	
	
0.0	1.190	0.0	1.148
0.5	1.285	0.5	1.253
1.0	1.406	1.0	1.389
2.0	1.527	2.0	1.521
3.0	1.632	3.0	1.632
4.0	1.782	4.0	1.805
5.0	1.918	5.0	1.955
6.0	2.016	6.0	2.069

$9.\frac{\text{Wt methane}}{\text{Wt air}+O_{2}}=0.070$		$10.\frac{\text{Wt methane}}{\text{Wt air}+O_{2}}=0.072$	
	
0.0	1.094	0.0	1.017
0.5	1.194	0.5	1.157
1.0	1.347	1.0	1.304
2.0	1.490	2.0	1.458
3.0	1.680	3.0	1.666
4.0	1.816	4.0	1.827
5.0	1.983	5.0	1.992
6.0	2.099	6.0	2.124

## Footnotes

1 Figures in brackets indicate the literature references at the end of this paper.
